# Vicks Floral Guide, 1890

**Published:** 1890-03

**Authors:** 


### Bibliographical.

VICKS FLORAL GUIDE, 1890.—From early life-time we have loved flowers, and one of the first things we did on getting a home of our own was to lay out a flower garden. We believe their influence has always been helpful, and that we are better to-day for their beauty and their fragrance.

If every home had its flowering shrubs, and its well-kept borders in which the rose bloomed and the carnation luxuriated, if the morning-glory nodded its sunrise welcome to each vigorous boy and the beautiful helena honeysuckle filled with fragrance the evening chamber of each beautiful girl, there would be fewer sons and daughters going to the bad, and many more sons holding to the farm and making glad the lessening days of the dear old father and mother who watched with eager interest their youthful steps.

We have from year to year called attention to these facts: we wish now to emphasize and give practical directions to them. We would like it if every home to which this paper comes would send to JAMES VICK, Rochester, N. Y., and get his FLORAL GUIDE, It will cost you 10 cents, which amount may be deducted from first order. It is one of our oldest friends. It came to us in our early housekeeping, before the elder Vick went home to enjoy the flowers of Paradise. We have kept up its acquaintance, but never has it come so uniquely beautiful as this year. It is unlike any floral catalogue, and will bring a sweet benediction into whatever home it enters. Flowers are earth's ornamentation from God's own hand, and the influence which they breathe is of divine origin.

